# Supplementary material for: The use and acceptability of preprints in health and social care settings: A scoping review
Source: PLoS One. 2023 Sep 15;18(9):e0291627. doi: 10.1371/journal.pone.0291627 (PMC10503772; doi:10.1371/journal.pone.0291627)
Supplement: S1 Appendix — (DOCX) [file pone.0291627.s001.docx]

**S1 Appendix: Search terms and keywords used**

| **Search topics/area** | **Search terms** |
| --- | --- |
| Preprints | Preprints  Version of record  Retraction |
| Open research | Open research  Open science  Open access |
| Research funding | Research awards  Grants  Fellowships  Research Funding  Funders  Funding organisations/organizations |
