# Supplementary material for: The use and acceptability of preprints in health and social care settings: A scoping review
Source: PLoS One. 2023 Sep 15;18(9):e0291627. doi: 10.1371/journal.pone.0291627 (PMC10503772; doi:10.1371/journal.pone.0291627)
Supplement: S1 Table — (DOCX) [file pone.0291627.s003.docx]

**S1 Table. Scopus and Web of science searches**

| **Scopus search (24/08/2022)** | **Results** | **Web of Science search (24/08/2022)** | **Results** |
| --- | --- | --- | --- |
| TITLE-ABS-KEY (preprints)  (TITLE-ABS-KEY (open PRE/1 research) OR TITLE-ABS-KEY (open PRE/1 science) OR TITLE-ABS-KEY (open PRE/1 access))  (TITLE-ABS-KEY (grant PRE/1 awards) OR TITLE-ABS-KEY ( research PRE/1 awards ) OR TITLE-ABS-KEY ( research PRE/1 funding ) OR TITLE-ABS-KEY ( funding PRE/1 organi* ) )  **(TITLE-ABS-KEY ( preprints ) ) AND ( ( TITLE-ABS-KEY ( open PRE/1 research ) OR TITLE-ABS-KEY ( open PRE/1 science ) OR TITLE-ABS-KEY ( open PRE/1 access ) ) )**  **(TITLE-ABS-KEY (preprints) ) AND ( ( TITLE-ABS-KEY ( grant PRE/1 awards ) OR TITLE-ABS-KEY ( research PRE/1 awards ) OR TITLE-ABS-KEY ( research PRE/1 funding ) OR TITLE-ABS-KEY ( funding PRE/1 organi* ) OR TITLE-ABS-KEY ( fund* ) ) )** | 4,720  46,476  10,861  **242**  **212** | #1 TS=(preprints)  #2 open research (Topic) or open science (Topic) or open access (Topic)  #1 AND #2  #3 grant awards (Topic) or research awards (Topic) or research funding (Topic) or funding organi* (Topic)  #1 AND #3  **#1 AND #2 and Other or Article or Review Article or Editorial Material or Other or Article or Review Article or Editorial Material or Meeting or Unspecified or Data Study or News or Early Access or Letter or Abstract or Patent or Data Paper or Reference Material (Document Types)**  **#1 AND #3 and Other or Article or Unspecified or Review Article or Data Study or Editorial Material or Meeting or News or Letter (Document Types)** | 9,847  1,686,411  2,814  239,219  388  **1,276**  **100** |
