# Supplementary material for: The use and acceptability of preprints in health and social care settings: A scoping review
Source: PLoS One. 2023 Sep 15;18(9):e0291627. doi: 10.1371/journal.pone.0291627 (PMC10503772; doi:10.1371/journal.pone.0291627)
Supplement: S2 Table — (DOCX) [file pone.0291627.s004.docx]

**Table S2: Full details of the 98 included articles**

| ***Authors*** | ***Year*** | ***Country - Main contributor*** | ***Source type*** | ***Publication and/or grant awards?*** | ***Key focus and highlights of the evidence relevant to preprints (excerpts from publications)*** |
| --- | --- | --- | --- | --- | --- |
| Abbasi, K. | 2018 | Unknown | Journal (Editorial) | Publications | Preprints have entered medical science. Publisher supports authors who wish to post their articles on preprint archives. Publisher in favour of open science dialogue; do not request open-access fee unless included in grant costs. |
| Alwine, J. C., Enquist, L. W., Dermody, T. S., & Goodrum, F. | 2021 | USA | Journal (Editorial) | Publications | Open-access as a business model is challenging (e.g., authors responsible for the cost of open-access publishing). Maintaining journalistic standards, integrity in scientific publishing and access are key aspects of publishing. |
| Avissar-Whiting, M., Bakker, C., Heckner, H., Massip, S., Schneider, J., et al | 2021 | USA | Journal (Editorial) | Publications | The use of preprints encourages greater transparency in the production/use of science and broad dissemination of scientific information and data reshapes how science is communicated and evaluated in scholarly communications. |
| Baffy, G., Burns, M. M., Hoffmann, B., Ramani, S., Sabharwal, S., et al | 2020 | USA | Journal (Editorial) | Publications | Scholarly communication is changing by complex digital tools (e.g., online access, social media, big data management). Current trends in scientific publishing may require new strategies from the academic and medical author community to protect enduring values and embrace promising developments. |
| Barbour, V. | 2021 | Australia | Journal (Editorial) | Publications | COVID-19 pandemic has exacerbated problems in scholarly communication: worsening participation by women and unequal distribution of funding globally. Uncertainty in preprints on how to support trust in research publications within academic and public communities. |
| Baždarić, K., Vrkić, I., Arh, E., Mavrinac, M., Marković, M. G., et al | 2021 | Spain | Journal (Original research) | Publications | Lack of validated questionnaires to measure attitudes towards open peer review, open data and use of preprints influence scientists' engagement with those practices. The development of Attitudes towards Open data sharing, preprinting, and peer-review (ATOPP) attempts to address that gap. |
| Bell, S. C., Flume, P. A., & Castellani, C. | 2020 | Australia | Journal (Editorial) | Publications | Preprint publishing has been criticised for jeopardising scientific integrity by publishing material without thorough and expert peer review. The proportion of published scientific papers that are pre-published is still low (<5%), yet the growth in the past five years confirms that this practice is being rapidly adopted by the broader scientific community. Whilst most journals accept formal submissions of preprinted research, some do not. Authors should carefully consider this when planning pre-print publishing of their work. |
| Berg, J. (2017) | 2017 | USA | Journal (Editorial) | Publications | The publisher encourages authors to discuss with their editors any postings to other servers and encourage editors to become involved in discussions about preprint-related issues. Interactions with the press related to preprints has become one challenging area. Preprints have the potential to address the challenges faced by early carer researchers, to some degree. The U.S. National Institutes of Health released a policy earlier this year that explicitly encourages the use of preprints and other “interim” research products. |
| Bernard, R., Weissgerber, T. L., Bobrov, E., Winham, S. J., Dirnagl, U., et al | 2020 | Germany | Journal (Correspondence) | Publications | Use of formats such as preprints in publishing findings. These alternative formats have the potential to reduce publication bias, but many scientists are unaware that these formats exist and don’t know how to use them. Expanding 'file drawers' of unpublished data may create problems for researchers, scientific community and public. |
| Bourne, P. E., Polka, J. K., Vale, R. D., & Kiley, R. | 2017 | USA | Journal (Editorial) | Publications and grants | Preprint servers include an uneditable timestamp indicating when the article was made public, which is usually within 24 hours of submission. This date, along with the preprint itself, is made open access (see Rule 2), and thus, anyone (using any internet search engine) can determine the order of priority relative to other published work or, indeed, other preprints. |
| Bosman, J., de Jonge, H., Kramer, B., & Sondervan, J. | 2021 | Netherlands | Journal (Review) | Publications | Explores options to further open access in the Netherlands from 2021. Goal of Dutch government to reach 100% open access to publicly funded research by 2020. There is a need to look at qualitative and quantitative aspects of open access. |
| Bozzato, V., Gnoato, M., Vilia, A., & Apostolico, M. | 2021 | Italy | Journal (Perspective) | Publications | Analyses the importance of protection of intellectual property (IP) in biomedical scholarly publications, both for the author’s reputation and the dissemination of scientific knowledge. |
| Brainard, J. | 2020 | USA | Journal (Perspective) | Publications | Preprints can be considered a valid scientific output from a research project. Quality of reporting in preprints is largely equivalent to that of peer-reviewed articles and supports researchers' job and grant applications. |
| Brierley, L., Nanni, F., Polka, J. K., Dey, G., Pálfy, et al | 2021 | UK | Journal (Original research) | Publications | One argument against the use of preprints suggests that preprints are less reliable than peer-reviewed papers as their conclusions may change in subsequent versions of the publication. Such concerns were amplified during the COVID-19 pandemic as preprints were used to shape policy and influence public opinion via coverage in social and traditional media. One implication of this hypothesis is that the peer review process is trusted to correct errors in studies and improve reproducibility, leading to significant differences between preprints and published versions |
| Callaway, E. | 2017 | Unknown | Journal (Perspective) | Publications | In February 2017 ASAPbio issued a funding call to build a central preprint site. Several leading funders announced their support for this (including NIH & the Wellcome trust). Support from funders send a message to the science community encouraging the use of this kind of communication. |
| Carà, P. D., Ciriminna, R., & Pagliaro, M. | 2017 | Italy | Journal (Editorial) | Publications | In Chemistry, attempts by public funding agencies to encourage researchers receiving public money to publish their research findings as open access articles have not been too successful. |
| Chaleplioglou, A., & Koulouris, A. | 2021 | Greece | Journal (Editorial) | Publications | Explores the scope and policies of the existing preprint papers platforms in different academic research fields. Preprints on an openly accessible server, as a citable object, represents an accelerated approach of academic communication. COVID-19 showed the importance of preprints as a form of scientific communication with nearly 40% of scientific publications released within the first 4 months after the disease outbreak hosted in preprint servers. Quick retraction of a faulty preprint COVID-19 paper within three days clearly demonstrated that the scientific community operates as the ultimate gate keeper of scientific integrity. Use of screening procedures on preprint servers is needed. |
| Chiarelli, A., Johnson, R., Pinfield, S., & Richens, E. | 2019 | UK | Journal (Original research) | Publications | Some challenges in the use of preprints. Preprint papers may be considered as 'prior publication' by some journals. There is concern about quality control/quality indicators in preprints. Some advantages: avenue to make research work public in short-term projects where grant funding for publication is an issue. |
| Choi, Y. J., Choi, H. W., & Kim, S. | 2021 | USA | Journal (Editorial) | Publications | Preprints have been recognized as a primary and essential method to disseminate new findings faster than traditional publications. Therefore, it has become necessary for journals and editors to acknowledge these changes, prepare preprint policies, and notify authors accordingly. |
| Clayson, P. E., Baldwin, S. A., & Larson, M. J. | 2021 | USA | Journal (Original research) | Publications | Barriers to accessing scientific findings contribute to knowledge inequalities due to financial resources and decrease the transparency and rigour of scientific research. |
| COPE Council | 2018 | UK | Journal (Perspective) | Publications | An important element for consideration relates to the licensing of material made available via preprints. A preprint platform may require authors to post the manuscript under a particular licence, which may conflict with the licence or copyright transfer agreements that may ultimately be required by a journal where the author intends to publish the work. Licensing is also a consideration in situations where a researcher may wish to self-archive the paper via a preprint platform after having published the work in a peer-reviewed journal. Some preprint platforms such as preprints.org note that preprints cannot be removed except in exceptional circumstances involving misconduct or legal concerns, but other servers may allow removal of content at the author’s request. |
| Eglen, S. J., Mounce, R., Gatto, L., Currie, A. M., & Nobis, Y. | 2018 | UK | Journal (Editorial) | Publications | Reproducibility crisis: key findings in publications are either not independently verified or fail verification when it is attempted. Publishing systems should take some responsibility for low levels of reproducibility as authors feel pressured to publish to avoid being scooped. Suggestions to promote reproducibility: pre-registration papers, stronger data sharing policies, reproducible manuscripts, replicability studies. |
| Ettinger, C. L., Sadanandappa, M. K., Görgülü, K., Coghlan, K. L., Hallenbeck, K. K., et al | 2022 | USA | Journal (Review) | Publications | Preprinting a manuscript involves a discussion among all co-authors, and early career researchers are often not the decision-makers. Therefore, early career researchers may find themselves in situations where they are interested in depositing a preprint but are unsure how to approach their co-authors or advisor about preprinting. Early career researchers benefit from posting preprints as they are shareable, citable, and prove productivity. |
| Eysenbach, G. | 2019 | Canada | Journal (Editorial) | Publications | Innovative open access model: papers will be published on preprint servers first, with 'overlay' journals then competing to peer review and publish peer-reviewed 'versions of record' of the best papers. |
| Ferreira, C. M., & Serpa, S. | 2018 | Portugal | Journal (Editorial) | Publications | Students/postdocs stay in training programs longer to publish, recent work is invisible to grant and award committees as well as colleagues; peer review is based on a small number of opinions; lack of transparency in the review process creates confusion about the priority of discovery. |
| Flanagin, A., Fontanarosa, P. B., & Bauchner, H. | 2020 | USA | Journal (Editorial) | Publications | Whether preprint posting and rapid dissemination of non–peer-reviewed reports of medical research that could have important clinical implications and consequences help achieve the goal of improving health outcomes for patients without causing harm remains uncertain. There is a general assumption that more rapid access to information will improve patient outcomes—the goal of research in clinical medicine. However, it is quite clear that in some countries, information from social media and preprint servers has been used by politicians and physicians to advocate for treatments, which is not the role of preprints communication. |
| Fleming, J. I., & Cook, B. G. (2022) | 2022 | USA | Journal (Review) | Publications | The lack of clarity from journals and publishers may deter some researchers from preprinting their work. Due to no peer-review, preprints may have flawed methods/biased results. Publishing Open Access (OA) can be time-consuming (finding the journal/publisher policy, identifying an appropriate preprint repository, signing up for the repository, formatting the preprint, copywriting the preprint and publishing online). |
| Fry, N. K., Marshall, H., & Mellins-Cohen, T. (2019) | 2019 | UK | Journal (Editorial) | Publications | Peer review: no formal peer review prior to posting preprints, but articles effectively available for scientific community to see and comment on. Publisher policies may vary in accepting preprints as manuscript submissions. Sustainability: funding for preprint servers is from non-profit agencies and concerns have been raised regarding sustainability and archiving costs. |
| Galbán Rodríguez, E. (2019) | 2019 | Cuba | Journal (Editorial) | Publications | Preprints as an alternative to underreporting/incomplete or misleading reporting by providing the first finished compilation of the protocol-summary results and final datasets. |
| George, C. H., Alexander, S. P. H., Cirino, G., Insel, P. A., Izzo, A. A., et al | 2021 | UK | Journal (Editorial) | Publications | BJP policy 2017 supports preprints. Policy was reviewed post-COVID-19 due to the increasing number of articles being submitted that cite preprints. |
| Gupta, L., Gasparyan, A. Y., Misra, D. P., Agarwal, V., Zimba, O., et al | 2020a | Korea | Journal (Original research) | Publications | COVID-19 pandemic has led to a large volume of publications, preprints, and retractions in a short amount of time. Social media has been identified as the most important source of information as well as misinformation regarding researchers/clinicians/academics' attitudes on preprints. Divided opinion on preprints for changing practice and retraction rates during the pandemic and perceived risk of plagiarism. |
| Gupta, L., Gasparyan, A. Y., Zimba, O., & Misra, D. P. | 2020 | Germany | Journal (Original research) | Publications and grants | Study that investigated the effect of competitive project funding on researchers’ publication outputs. Accessibility/dissemination as measured by publication of preprints. "... consider preprints which have become an important mode of disseminating research results quickly but received so far no attention in the research of funding effects." Past research performance has been found to be a strong predictor of grant success where peer-reviewed articles matter more than preprints. |
| Grant, S., Wendt, K. E., Leadbeater, B. J., Supplee, L. H., Mayo-Wilson, E., et al | 2022 | USA | Journal (Perspective) | Publications and grants | Preprints allow researchers to have direct access to findings and make evidence timelier. Funders can implement policies to promote transparency, openness, and reproducibility of research they fund. Funders could request researchers be transparent about their procedures and share all products of their funded research. DORA: suggests considering the value and impact of research outputs beyond publications (e.g., datasets, software, and code) when evaluating the scientific productivity of grant applications. |
| Green, T. | 2019 | France | Journal (Editorial) | Publications | A digital transformation of scholarly communications based on internet-era principles is needed if OA is to succeed. Considers two-step publishing. |
| Heyard, R., & Hottenrott, H. | 2021 | Germany | Journal (Original research) | Publications and grants | The study investigated the effect of competitive project funding on researchers’ publications and outputs. Findings show researchers with a successful grant publish on average 1.2 articles and about one additional preprint in the following year. Researchers’ articles receive 1.7 citations more than articles from the study control group. Preprints do not undergo the peer-review process but help researchers communicate their results to their community and to secure priority of discovery. |
| Howat, A. M., & Clark, J. | 2021 | UK | Journal (Editorial) | Publications | Adopt transparent peer review model and an open data policy, with an underlying principle of 'as open as possible, as closed as necessary' |
| Hurst, P., & Greaves, S. | 2021 | UK | Journal (Review) | Publications | COVID-19 research published more quickly by encouraging experts to join a reviewer pool to quickly review preprint and journal submissions. At the same time the peer-review system was proving too slow. Pandemic confirmed that use of preprints is important in scholarly communication. Media/government quoting unreviewed preprints and journal articles were retracted. |
| Ibragimova, I., & Phagava, H. | 2022 | USA | Journal (Perspective) | Publications | Advice: media should not report on preprints, and institutions should not promote preprints to the media. Researchers to check journal publication policy prior to depositing their preprints, as the compatibility of publishing a preprint in a peer-reviewed journal could meet three types of restrictions: server type, paper version (posting of the revised manuscript) and licence of preprint distribution. |
| Itani, D., Lababidi, G., Itani, R., El Ghoul, T., Hamade, L., et al | 2022 | Lebanon | Journal (Original research) | Publications | A study highlighted the importance of having clear policies for reporting funding and Conflict of Interest (COI) in preprint servers. Researchers (preprints authors) must report comprehensive COI and funding sources in the preprints versions of manuscripts to minimise changes between pre-publication and post publication versions. |
| Kaiser, J. | 2017 | USA | Journal (Perspective) | Publications | Some worry that early sharing risk competitors stealing their data or ideas, or rush to publish similar work. Others predict that preprint servers will become a time sink, as scientists spend hours trying to sift through an immense mishmash of papers of various quality. And some researchers fear that easy, rapid publication could foster preprint wars—in which the findings in one preprint are quickly attacked in another, sometimes within hours. Such online squabbles could leave the public bewildered and erode trust in scientists. Some researchers avoid posting to preprint archives because they believe that the screening and sorting performed by traditional journals serves readers well. |
| Kaiser, D. E., & Oliveira Crossetti, M. | 2021 | Brazil | Journal (Editorial) | Publications | Challenges to preprints use should be defined in the fields of Nursing and Health Sciences: partial information from the production of knowledge can have an impact on professional practice, as well as an impact on people’s and collective health. Therefore, a preprint demands quality and an important theoretical discussion of potential and limits previous to the adoption of the model. |
| Kirkham, J., & Moher, D. | 2018 | UK | Journal (Original research) | Publications | Concerns about the peer review process and the quality of the articles that were published under the F1000 model. Some also found that the peer review process took longer than standard journals because there was more emphasis on the authors rather than editors to find peer reviewers. There was also a sense that there was the potential for an article to become caught up in the process, immediate publication meant that there was limited scope to remove or submit elsewhere if peer reviewers could not be found or existing reviewers failed to provide subsequent reviews. |
| Klebel, T., Reichmann, S., Polka, J., McDowell, G., Penfold, N., et al | 2020 | Austria | Journal (Original research) | Publications | Lack of clarity in preprints policies in major journals regarding disclosure of reviewers’ identities to authors only, information on whether preprints can be posted or not, the type of peer review used (double blind, single blind, not blinded, or other), position on co-reviewing, citation of preprints, publication of reviewer identities, and practices of open peer review. |
| Kleinert, S., Horton, R., & Editors of the Lancet family of journals. | 2018 | USA | Journal (Commentary) | Publications | Preprints should not be used for clinical decision making or reporting of research findings to a lay audience without indicating that this is preliminary research that has not been peer reviewed. |
| Koerber, A. | 2021 | USA | Journal (Editorial) | Publications | We are learning to navigate a situation in which tidbits of scientific data can easily circulate and become understood as gospel truth or, just as easily, be dismissed as fake, depending on the audience who happens to receive them. It is increasingly difficult now for scientists to just work quietly in the laboratory and then, many months later, publish their findings in peer-reviewed journals from which journalists and other professional communicators can then translate these findings for public audiences. Conspiracy theories emerge from the same open-science practices that encourage early sharing of research/science. |
| Kowalczyk, O. S., Lautarescu, A., Blok, E., Dall’Aglio, L., & Westwood, S. J. | 2022 | UK | Journal (Perspective) | Publications | Identifying and keeping track of open research funding opportunities is challenging. Examples of funding opportunities supporting or rewarding open research include NIH, UKRI, Wellcome Trust. For open research to become the norm, further engagement/support must come from senior academics given their involvement in supervision, peer-review, journal editing, hiring, and informing institutional policies. |
| Kullmann, D. M. | 2017 | UK | Journal (Editorial) | Publications | Publication of underpowered experimental studies probably contributes to the poor success rate of clinical translation, and this crisis would most likely be worsened if preprint publication became the standard model of communication of scientific advances. |
| Kwon, D. | 2020 | USA | Journal (Commentary) | Publications | Not all researchers might want to adopt preprints. eLife will adopt a 'publish, then review' policy, and will make all its peer-review reports publicly available. eLife only reviews and publishes papers that have already been posted on a preprint server, such as bioRxiv, medRxiv or arXiv Submitted papers that are not already on preprint servers will be posted on bioRxiv or medRxiv. |
| Lee, A. Y. S., & Lin, M. W. | 2020 | Australia | Journal (Commentary) | Publications | Publications of hastily penned observations may mislead and do more harm than good |
| Leopold, S. S., Haddad, F. S., Sandell, L. J., & Swiontkowski, M. | 2019 | USA | Journal (Editorial) | Publications | Preprint servers offer the benefits of durability, speed of posting, and easy access. Other advantages include receiving feedback on work from other researchers and disseminating results without barriers such as journals’ subscription paywalls or delays with peer review. |
| Lombardi, S. | 2020 | Italy | Conference Proceeding | Publications | With speed comes errors as observed in COVID-19 preprint publications that were withdrawn. Some preprints will never become published articles. |
| Machin-Mastromatteo, J. D., Tarango, J., & Romo-González, J. R. | 2021 | Mexico | Journal (Original research) | Publications | Changes could occur to the traditional peer review process, which is opaque and errors that may escape it might take months or years (if ever) to be identified and then cause the article to be retracted. The quality conditions required to publish manuscripts, to evaluate them with transparency and strong scrutiny, have always been accepted as necessary. The pandemic has strengthened this position and drawn further attention to their flaws. |
| McBee, M. T., Makel, M. C., Peters, S. J., & Matthews, M. S. | 2018 | USA | Journal (Commentary) | Publications | Publishing companies preprint policies vary. There may be confusion over the version of record if the manuscript is available in several different forms. For example, if the print is the originally submitted manuscript but the published article was revised, there may be important differences between the two, although this has been studied and changes between preprint versions and version of record are not great. The existence of multiple versions can split citations across the different versions, thus pointing future readers to different texts and potentially harming the research team's h or i10 indices of scholarly impact. |
| McGuinness, L. A., & Sheppard, A. L. | 2021 | UK | Journal (Original research) | Publications | Requiring that authors submit a data availability statement is a good first step, but is insufficient to ensure data availability, especially in preprints since the analysis of 1 year worth of preprints data show better coverage of data availability in version of record (Data availability statements more frequently described open data on publication when the journal mandated data sharing (open at preprint: 33.3%, open at publication: 61.4%) compared to when the journal did not mandate data sharing (open at preprint: 20.2%, open at publication: 22.3%)). |
| Musunuri, S., Sandbrink, J. B., Monrad, J. T., Palmer, M. J., & Koblentz, G. D. | 2021 | USA | Journal (Original research) | Publications | Research associated with the greatest misuse potential constitutes “dual-use research of concern” (DURC), which the U.S. National Institutes of Health defines as “life sciences research that, based on current understanding, can be reasonably anticipated to provide knowledge, information, products or technologies that could be directly misapplied to pose a significant threat with broad potential consequences to public health and safety.” |
| Moshontz, H., Binion, G., Walton, H., Brown, B. T., & Syed, M. | 2021 | USA | Journal (Commentary) | Publications | Guide to help psychological scientists to post preprints and manage them across the publication pipeline. Posting preprints is a free and legal way that researchers can use to make their work open access. |
| Mwangi, K. W., Mainye, N., Ouso, D. O., Esoh, K., Muraya, A. W., et al | 2021 | Kenya | Journal (Original research) | Publications | Open Science and preprints in Kenya (and other countries in Africa). Out of 20,069 papers downloaded from BioRXiv, only 18 had Kenyan authors, a majority of which had international (16) collaborations. This may suggest poor uptake of the use of preprints among Kenyan researchers. The findings in this study highlight the state of open science in Kenya and challenges facing its adoption and practice while bringing forth possible areas for primary consideration in the campaign toward open science. |
| Nature Communications. (2017) | 2017 | Unknown | Journal (Editorial) | Publications | Promoting preprints under peer review represents for us another small step towards a more open and transparent peer review process in which preprints and the community’s feedback on them play an important part. There is recognition peer review is a lengthy process. Preprint servers allow authors to publish/disseminate without delay. |
| Nishioka, C., Färber, M., & Saier, T. | 2022 | Germany | Journal (Original research) | Publications and grants | Citation bias exists and it is more severe in case of preprints. Larger citation inequalities in preprints than in published versions indicating that the author affiliations might influence the readership and the perception of preprints.  As preprints are on the rise, affiliation-based citation bias is an important topic not only for authors (e.g., when deciding what to cite), but also to people and institutions that use citations for scientific impact quantification (e.g., funding agencies deciding about funding based on citation counts). |
| Pagliaro, M. | 2021 | Italy | Journal (Editorial) | Publications | By making their work freely and immediately accessible on the internet first in the form of preprints and subsequently in the form of peer reviewed journal articles in OA or paywalled journals, chemistry scholars too will rapidly reap the benefits of open science already demonstrated in closely related disciplines (life sciences and physics) in terms of enhanced citations, media attention, collaborations, job, and funding opportunities. |
| Jui-An Pan, S. | 2022 | Taiwan | Journal (Commentary) | Publications | Currently, the mechanism for the publication of preprints has gradually trended toward the form of journal publication, which has increasingly blurred the boundary between preprints and peer-reviewed articles. Although the practice of publishing preprints has been gaining popularity, there is an absence of principle-based regulations to achieve publication ethics. Numerous aspects of enhancing the accountability and transparency of scholarly publishing require considerable effort on the part of both authors and preprint servers (e.g., transparency in the time sequence of publication between preprints and subsequent peer-reviewed journal articles; most preprint servers operate without teams that have expertise in different disciplines). |
| Peiperl, L., & on behalf of the Plos Medicine Editors. | 2018 | USA | Journal (Commentary) | Publications | As preprint servers for clinically focused research become more widely available and preprint posting becomes more routine and more closely integrated with journal publishing, editors of medical journals should join the conversation about best practices for preprint sharing in medical research. As opportunities for preprint sharing advance, medical journals should endeavour to ensure that the resulting changes benefit human health and support the public understanding of medical research. |
| Pearse, R. M., Ackland, G. L., Asai, T., & Hemmings, H. C., Jr. | 2021 | UK | Journal (Commentary) | Publications | In a public emergency, it becomes urgent to complete medical research and place the findings expeditiously into the public domain after expert peer review so that new findings can be used to improve patient care as soon as possible. The process of peer review is often a slow process but is essential to ensure that changes in patient care are informed by careful and definitive research. Journal editors must balance the potentially competing goals of immediacy and quality control. |
| Peretz, F., Bonini-Vuillod, J., Grivaux, M., Duracinsky, M., & Chassany, O. | 2021 | France | Journal (Commentary) | Publications | The media coverage of three COVID-19 articles analysed, regardless of their quality and the level of proof of their conclusions, had an impact on individual behaviour and public decision’s ability of users of preprints manuscripts to judge the scientific quality of manuscripts. Readers are not always scientists, well trained in clinical research, knowledgeable of good clinical practices ensuring ethical and scientific quality, or the reasoning and methodology of the trials; few are able to verify the quality of the study and its methodological biases, especially since this verification requires multiple cross-checks and takes time. |
| Polka, J. | 2017 | USA | Journal (Original research) | Publications | Competition among researchers to squeeze as much impact from every publication as possible. It takes PhD students longer to graduate and publish their first research publication. Publications today include more data than they did over 30 years ago. Preprints are beneficial for researchers working in highly competitive fields. |
| Poremski, D., Falissard, B., Fegert, J., Witt, A., Ordóñez, A. E., et al | 2019 | Singapore | Journal (Perspective) | Publications | Preprints allow authors to receive prompt feedback from a larger community of colleagues than two or three experts who might typically review their manuscript. Increases visibility and speed in disseminating findings and help counterbalance the effects of publication bias. Authors are advised to review journal policy before uploading their work to a preprint repository. |
| Price, R., & Ozkan, Y. | 2021 | UK | Journal (Original research) | Publications | High-demand placed on journals during the COVID-19 pandemic. The presence of exclusively non-peer reviewed material from January to March (during lockdowns periods in some countries) suggests that demand could not be met by journals in this period, and the sector supported this with enhanced preprint services for authors. |
| Raynaud, M., Zhang, H., Louis, K., Goutaudier, V., Wang, J., et al | 2021 | France | Journal (Original research) | Publications | Emergency measures and rapid adaptation by healthcare workers is important and scientific communication should be promoted. Nevertheless, the authors urge healthcare researchers and practitioners to evaluate medical publications with appropriate skepticism despite the sense of urgency that the pandemic generated, and to bear in mind that high standards of research are needed to make progress in controlling a pandemic. |
| Sarabipour, S., Debat, H. J., Emmott, E., Burgess, S. J., Schwessinger, B., et al | 2019 | USA | Journal (Commentary) | Publications and grants | ECRs face a changing academic landscape, including the increased interdisciplinarity of life sciences research, expansion of the researcher population, and consequent shifts in employer and funding demands. |
| Schapira, M., The Open Lab Notebook Consortium, & Harding, R. J. | 2019 | Canada | Journal (Editorial) | Publications | Open laboratory notebooks: research scientists can share their research; including protocols, negative/positive results, online and in near real-time. |
| Schloss, P. D. | 2017 | USA | Journal (Editorial) | Publications | Preprinting in microbiology: preprints have emerged as a tool that microbiologists are using for disseminating new knowledge to peers and the public. Posting preprints can help to transparently recruit a more diverse pool of reviewers prior to submitting a journal for formal peer review. |
| Sever, R., Eisen, M., & Inglis, J. | 2019 | USA | Journal (Perspective) | Publications | The incumbent system of research communication has two main problems. It is slow: it typically takes around a year for a manuscript submitted to a research journal to be peer-reviewed, accepted, and published online. And research outputs are not widely accessible: many papers are published in journals available exclusively via subscription, which may restrict their readership to researchers in certain universities and countries. |
| Serpa, S., Sá, M. J., Santos, A. I., & Ferreira, C. M. | 2020 | Portugal | Journal (Perspective) | Publications | Academic editors will have to be able to cope in a world of increasing open access dominance, with challenges, such as (i) journal indexing and metrics (ii) the increasing pressure for articles to explicitly indicate their “practitioner impact” (Hughes et al., 2018, p. 2); (iii) the growing preprint publication with manuscripts not previously peer-reviewed before being made publicly available; (iv) the presence of references of articles in social networks assessed through Altmetrics or similar indicators; (v) the mega-journals, which have a focus that covers a very large number of topics; and, finally, (vi) decolonise the international scientific publication, acknowledging that scientific quality is not present only in the Anglophone centre’s model and language. |
| Smart, P. | 2022 | UK | Journal (Perspective) | Publications | Research suggests that not all preprints are published in peer reviewed journals (or other accredited outlets), although it is impossible to accurately assess the number that are. **Acceptance**: while individual publishers and journal editors may still feel somewhat uncomfortable with preprints, there has been a general policy change, and many now accept these articles. **Encouragement**: moving further, several journals now actively encourage authors to preprint their articles—going one step beyond stating that preprints are accepted. Some journals even offer to upload articles to preprint servers on behalf of the authors following submission (e.g., PLOS) **Participation**: some journals have changed from “opt in” (i.e., “would you like us to upload your article onto the preprint server?”) to opt-out (i.e., “we will upload your article onto the preprint server unless…”). |
| Smith, J. A., & Sandbrink, J. B. (2022) | 2022 | UK | Journal (Perspective) | Publications and grants | A key difference compared to journal articles is that some preprint servers do not screen scientific articles before they are made publicly available. In one analysis, 68% provided some form of screening or moderation before the article was made public. Unlike publishing in particular journals, there is little incentive to post to a particular preprint server, so little reason not to select one that will immediately post the article. Whether this is important depends on the role that journals play in preventing or altering publication of research with potential for misuse. There are many routes to making information available publicly over which journals have no control, such as personal websites, news articles, or conference presentations. However, there are examples where journals and editors have been important in evaluating risks from publication. |
| Smyth, A. R., Rawlinson, C., & Jenkins, G. (2020) | 2020 | UK | Journal (Perspective) | Publications | Debate over effectiveness of peer-review. Peer-review process causes delay in making findings publicly available. No correlation between the number of rounds of manuscript review and revision and subsequent citation count for the paper. Preprints do not undertake peer review but restricts scrutiny to basic screening and legal checks. Rush to publication is an editorial and scientific mistake. |
| Soderberg, C. K., Errington, T. M., & Nosek, B. A. (2020) | 2020 | USA | Journal (Original research) | Publications | A survey to gather information about cues that could be displayed on preprints to help researchers assess their credibility. Understanding whether and how heuristic cues can be used for initial assessments of credibility is important for meeting the promise that preprints hold for opening and accelerating scholarly communication. |
| Sopinka, N. M., Coristine, L. E., DeRosa, M. C., Rochman, C. M., Owens, B. L., et al | 2020 | Canada | Journal (Perspective) | Publications | Media may sensationalise research before it is vetted as sound science (Tennant et al. 2017), or worse, policy decisions may be based on research where flaws have not yet been corrected during peer review. Peer review serves a vital role in preserving the integrity of scientific information (Mulligan et al. 2013); it will continue to do so for the future. The present lack of diversity and inclusion within science limits the capacity for science to reach diverse audiences through diverse mediums (Puritty et al. 2017). Only when science itself is a diverse and inclusive enterprise, will the discipline be capable of maximising discovery and innovation (Valantine and Collins 2015) and effectively communicating to diverse users in diverse ways (Puritty et al. 2017). |
| Subramanian, K., Nalli, A., Senthil, V., & Bhat, A. | 2021 | China | Journal (Original research) | Publications | While preprints are not a substitute for peer-reviewed publications, they could be considered in case faster dissemination of the research is needed for global healthcare. Nevertheless, citation of preprints might require guidance to prevent dissemination of flawed science. |
| Staines, H., & Martone, M. E. | 2018 | Unknown | Journal (Perspective) | Publications | Scholarly journals saw preprints as a threat to their business model. When journals moved online, so too did the letters to the editor. Many publications also added commenting functionality which made it simpler for researchers to share their opinions on articles and even to reply to the opinions of others. Researchers post these early versions of their results with the hope that reader input will improve or inform them, setting up an ideal opportunity for community interaction. With the support of preprint servers, who have indicated their willingness to integrate publisher-branded and -moderated layers, publishers can expand their community to make their branded layers visible on those sites as well. |
| Strcic, J., Civljak, A., Glozinic, T., Pacheco, R. L., Brkovic, T., & Puljak, L. | 2022 | Hungary | Journal (Original research) | Publications | It is not known how often authors that post their manuscripts in preprint servers also make their raw data available together with a manuscript, particularly in the case of a public health emergency. This study aimed to analyse data sharing statements and actual data sharing in articles about COVID-19 published in preprint servers medRxiv and bioRxiv. This study found that only a quarter of preprint articles on COVID-19, posted on bioRxiv and medRxiv, had a data/code sharing statement within the manuscript. Furthermore, among the preprint articles that reported that data were available somewhere (i.e., in the manuscript or online in a repository, etc.) |
| Teixeira da Silva, J. A. | 2017 | Japan | Journal (Editorial) | Publications | Ethical concerns preprints used as a poor science output: no peer-review, not scientifically vetted, not verified for potential errors, flaws, or fraud. Preprints superficially selected by an advisory board and published quickly. Federation of American Studies for Experimental Biology: preprints would overburden an already overburdened peer pool but would also have a negative effect on rigour and reproducibility of research. Metrics considered an academic threat in preprints. |
| Teixeira da Silva, J. A. | 2018 | Japan | Journal (Perspective) | Publications | The underlying concern is that preprints might contain factually incorrect information. Preprints should be observed exclusively as a work in progress made open to the public for open feedback, either to improve the paper itself or the methodologies cited therein, but not to be mistaken with open peer review, which is a more formal and accountable process meant to detect errors prior to becoming a final citable and usable scholarly item. The risk of scooping intellectual ideas such as methods from a preprint is unlikely because a preprint offers time-sensitive evidence of an intellectual claim. |
| Teixeira da Silva, J. A. | 2020 | Japan | Journal (Editorial) | Publications | Preprints related to COVID-19 have been cited into academic literature, despite not being peer-reviewed or vetted by medical/other experts. Small number of preprints have been withdrawn/retracted. Strict ethical guidance are urgently needed for preprints, and preprint authors, in the case of misconduct, should face the same procedure and consequences as standard peer-reviewed academic literature. |
| Teixeira da Silva, J. A. | 2021 | Saudi Arabia | Journal (Editorial) | Publications | The focus of the discussion is no longer ‘whether preprints should be cited’ but rather ‘how preprints should be cited’ to reduce the risks to the integrity of the literature and human health. Preprint servers should instill a basic, but rigorous, level of screening and quality control, at a minimum to establish open-science policies to ensure that basic ethical requirements are met. |
| Teixeira da Silva, J. A. | 2022 | Japan | Journal (Editorial) | Publications | Version control: preprints can be corrected if version is updated and is limited only to minor edits/corrections. Queries why preprint servers are not COPE members. Preprints should be cautiously used, screened, and cited as they represent incompletely scrutinised documents that have not been validated by peers through traditional peer-review. |
| Teixeira da Silva, J. A., & Dobránszki, J. | 2019 | Japan | Journal (Original research) | Publications | Given the increasing use of preprints, the objective of this study was to use the Sherpa/ RoMEO database which offers a summary of the preprint policies of publishers it indexes, to assess how 14 scientific publishers use or allow the use of preprints during their publishing process. Policies on preprints need to be clear, consistent, and transparent. Preprints are to some extent an expression of the failure of traditional biomedical publishing. Therefore, preprints have been marketed as a solution to the replication crisis and serve as a replication-fixing tool by allowing biologists to challenge published results. |
| Teixeira da Silva, J. A., Tsigaris, P., & Al-Khatib, A. | 2019a | Japan | Journal (Editorial) | Publications | Open access has become explored by low-tier 'predatory' entities that might offer weak or no quality control in the form of peer-review, editorial oversight, and post-publication review (PPPR). PPPR: effective strategy to identify flaws in published literature. |
| Tennant, J. P., Crane, H., Crick, T., Davila, J., Enkhbayar, A., et al | 2019 | France | Journal (Perspective) | Publications | Will preprints get your research ‘scooped’? - To the best of our knowledge, there is virtually no evidence that ‘scooping’ of research via preprints exists, not even in communities that have broadly adopted the use of the arXiv server for sharing preprints since 1991. Does approval by peer review prove that you can trust a research paper, its data, and the reported conclusions? Even though experts often criticise peer review for several reasons, the process is still often considered the “gold standard” of science. Occasionally however, peer review approves studies that are later found to be wrong, and rarely deceptive or fraudulent results are indeed discovered prior to publication. By failing to effectively communicate that peer review is imperfect, the message conveyed to the wider public is that studies published in peer-reviewed journals are “true” and that peer review protects the literature from flawed science.  Will the quality of the scientific literature suffer without journal-imposed peer review? Some have argued that without the filter provided by peer review, the literature risks becoming a dumping ground for unreliable results. |
| Triggle, C. R., MacDonald, R., Triggle, D. J., & Grierson, D. | 2022 | UK | Journal (Editorial) | Publications | Preprint servers made a great impact during COVID-19 pandemic. Preprint server articles do not appear in PubMed and may be considered a disadvantage; however, they do provide an avenue for progression from preprint server to peer-reviewed journal. We should ask: “Has this increase in quantity of both paper and journals reduced the quality of the published products?” |
| Verma, I. M. | 2017 | Canada | Journal (Editorial) | Publications | While publications await peer-review, preprints ensure research results are published in a timely fashion. Preprints allow authors to collect feedback and improve their work before submitting to peer-review, avoid duplicating research that others have already carried out, publish null-findings (may go unnoticed as due to insufficient interest from journals), influence study design, allow opportunities for collaboration. |
| Verma, A. A., & Detsky, A. S. | 2020 | Canada | Journal (Editorial) | Publications | Preprints aim to increase transparency, accelerate the exchange of ideas, and enhance collaboration. Preprints change the incentive structure of academic publishing and therefore may change behaviour and change culture as they offer a weak reward (preprint publication) for less effort than peer-review publication. |
| Watson, C. | 2022 | Australia | Journal (Commentary) | Publications | Despite the drawbacks there is little doubt that preprint publishing will stay, and the question becomes how to ensure its quality, transparency, and scientific responsibility, this is, how science will handle it. Journal publications are potentially dangerous if readers assume peer review equates to certified quality science. |
| Vlasschaert, C., Topf, J. M., & Hiremath, S. | 2020 | Canada | Journal (Perspective) | Publications | Preprints offer key critical advantages. For example, open access, easy feedback, and faster dissemination. Every crisis presents an opportunity, and COVID-19 is one with the potential to revolutionize and democratize the dissemination of scientific research. |
| Vuong, Q. H. | 2020 | Vietnam | Journal (Perspective) | Publications | Preprints reflect technological progress primarily in the lowering of costs and increasing the speed of product completion. However, self-publishing via preprint servers may threaten the quality of academic research |
| Wang, Y., Cao, Z., Zeng, D. D., Zhang, Q., & Luo, T. (2021) | 2021 | China | Journal (Original research) | Publications | The validity of the COVID-19 parameter estimations of the preprints was on par with that of peer-reviewed publications, and synthesized results of literatures could reduce the uncertainty and be used for epidemic decision-making. |
| Weissgerber, T., Riedel, N., Kilicoglu, H., Labbe, C., Eckmann, P., et al | 2021 | Germany | Journal (Editorial) | Publications | COVID-19 has thrust preprints into the spotlight, attracting attention from the media and the public, as well as from scientists. Preprints offer a unique opportunity to improve reporting. The Automated Screening Working Group aims to provide rapid feedback that may help authors of COVID-19 preprints to improve their transparency and reproducibility. It is feasible to conduct large-scale automated screening of preprints and provide rapid feedback to authors and readers. Recognition that automated tools are not perfect and cannot always determine whether a problem is relevant to a research paper. Some problems are too complex for automated tools to detect. Despite these limitations, automated tools can quickly flag potential problems and may complement peer reviews. |
| Yi, H. J., & Huh, S. | 2021 | Korea | Journal (Original research) | Publications | Preprints are still not actively used in Korea. Despite experiences with preprints not being widespread, respondents showed favorable attitudes towards preprints. More preprint policies should be accepted by editors in Korea. |
