# Supplementary material for: The use and acceptability of preprints in health and social care settings: A scoping review
Source: PLoS One. 2023 Sep 15;18(9):e0291627. doi: 10.1371/journal.pone.0291627 (PMC10503772; doi:10.1371/journal.pone.0291627)
Supplement: S3 Table — (DOCX) [file pone.0291627.s005.docx]

**S3 Table. Publishers and Journals preprints policy or guidance (in alphabetical order)**

| **Journal/publisher** | **Notes on Preprints** | **Source / Preprint server details (last accessed: 10/07/2023)** |
| --- | --- | --- |
| ASAPBio | List of preprint servers: policies and practices across platforms | [List of preprint servers: policies and practices across platforms – ASAPbio](https://asapbio.org/preprint-servers) |
| Biomed Central (BMC) | This service, called “In Review” links with the submission system used by BMC. As articles go through peer review, they are tracked, and the status of the article is reported against the preprint on ResearchSquare. | Linking service introduced onto the preprint server ResearchSquare: <https://www.researchsquare.com/publishers/in-review> |
| British Medical Journal (BMJ) | BMJ fully supports and encourages the archiving of preprints in any recognised, not-for-profit, preprint server. | [Preprints - BMJ Author Hub](https://authors.bmj.com/policies/preprints/) |
| Cambridge University Press | Platform for rapid, author-led publication and open peer review, including preprint policy. Encourages authors to include details of preprint posting, including DOI or other persistent identifier. | <https://www.cambridge.org/core/services/open-access-policies/open-access-journals/preprint-policy>  <https://www.cambridge.org/core/services/open-access-policies/open-access-journals/green-open-access-policy-for-journals> |
| eLife | Will only accept manuscripts that have already been made available on a preprint server | medRxiv or bioRxiv <https://elifesciences.org/articles/64910>  <https://elifesciences.org/inside-elife/00f2f185/elife-latest-preprints-and-peer-review> |
| Elsevier | First Look on SSRN is a place where journals identify and share content of interest prior to publication. The early stage research shared can include preprints, accepted papers and papers under consideration. | [first-look :: SSRN](https://www.ssrn.com/index.cfm/en/first-look/) <https://www.elsevier.com/connect/how-preprints-can-make-research-more-discoverable> |
| EMBO | Authors who upload their articles onto bioRxiv can request a review from the EMBO. editorial office. EMBO manages peer reviews to obtain two reviews which are posted against the article on bioRxiv. Once the reviews have been received, authors can revise and submit to one of 17 participating journals which use the reviews as the basis for accepting or rejecting the revised article [www.reviewcommons.org](http://www.reviewcommons.org)  Four of EMBOs journals also have scooping protection | This service is limited as it relies on the goodwill and the capacity of both EMBO editorial offices, and so is not ultimately scalable.  [www.reviewcommons.org](http://www.reviewcommons.org)  Four of EMBOs journals also have scooping protection |
| Emerald Group Publishing | Emerald will consider papers for publication that have been posted to a preprint server before they are submitted to Emerald. | [Prior publication – preprints, conference papers & theses \| Emerald Publishing (emeraldgrouppublishing.com)](https://www.emeraldgrouppublishing.com/publish-with-us/author-policies/pre-prints-conference-papers-policies) |
| F1000 Research | Now owned by Taylor & Francis, was the first journal to create a model that merged both the immediacy of the preprint server with the selectivity of journals. Submitted articles are published immediately after a quick evaluation, including a plagiarism check, etc. Reviewers are then invited, and their reviews are posted against the article. The reviewers make the publishing decision, and if there is sufficient consensus, the article is considered “Accepted” or “Rejected.”  F1000 Research offers a unique publishing platform, which like preprint servers offer immediate publishing but with the added advantage of post-publication peer review and eventual article indexing on a bibliometric database^(34)^ | If accepted, the article goes into various indexes, including Scopus and MEDLINE Authors can revise their articles at any point, and all revisions are linked to previous and subsequent versions— each undergoing the same check and review process. |
| JAMA Network journals | In 2019 changed its policy *“Public dissemination of manuscripts prior to, simultaneous with, or following submission to this journal, such as posting the manuscript on preprint servers or other repositories, will necessitate making a determination of whether publication of the submitted manuscript will add meaningful new information to the medical literature or will be redundant with information already disseminated with the posting of the preprint.”(9)*  To help encourage transparency, the JAMA Network also recommends that authors who cite preprints in their manuscripts indicate “preprint” in the citation in reference lists. | <https://jamanetwork.com/journals/jama/pages/instructions-for-authors?utm_campaign=articlePDF%26utm_medium=articlePDFlink%26utm_source=articlePDF%26utm_content=jama.2020.20674> |
| Lancet | Preprints intended for research use. Due to preprints not being peer-reviewed, they should not be used for clinical decision making or reporting of research to a lay audience without indicating is has not been peer-reviewed. All authors now ‘opt-out’ (we will upload your article onto the preprint server unless…) of having their manuscript uploaded to a preprint server, a move away from an ‘opt-in’ (would you like us to upload your article onto the preprint server?) | <https://www.thelancet.com/preprints>  All submissions are uploaded onto SSRN the preprint server owned by Elsevier^(6)^ <https://www.ssrn.com/index.cfm/en/>  <https://www.thelancet.com/journals/lancet/article/PIIS0140-6736(18)31125-5/fulltext> |
| Nature and Nature Medicine | In 2019, the Nature journals, including Nature Medicine, announced a move from support to encouragement of preprints and advise that authors can engage with news media about their preprint studies provided they explain that the study has not been peer reviewed and that findings could change. | <https://www.nature.com/nm/editorial-policies/preprints-conference-proceedings> |
| Oxford University Press | Authors of open access articles are entitled to deposit their original version or the version of record in institutional and/or centrally organized repositories and can make this publicly available immediately upon publication, provided that the journal and OUP are attributed as the original place of publication and that correct citation details are given. Authors should also deposit the URL of their published article, in addition to the PDF version. | <https://academic.oup.com/pages/self_archiving_policy_c> |
| PLOS | PLOS have accepted preprints since 1 May 2018. Some journals even offer to upload articles to preprint servers on behalf of the authors following submission. PLOS authors are asked if they have uploaded onto a preprint server and, if not, if they would like the journal to do this on their behalf.  In 2020, PLOS journals amended their policy toward preprints and publication embargoes, in that manuscripts previously posted as preprints and accepted for publication remain under a news embargo. | In this case, articles are uploaded onto bioRxiv or medRxiv <https://journals.plos.org/plosone/s/preprints> |
| SAGE | Advance: a SAGE preprints community allows researchers within the fields of humanities and social sciences to post their work online and free of charge. Advance welcomes a variety of preprint* types, including, but not limited to, original research, literature reviews, commentaries, and case studies.   Advance facilitates broad dissemination of ideas earlier in the research process and extends SAGE's mission of disseminating research on a global scale, in turn further supporting open science. | <https://uk.sagepub.com/en-gb/eur/preprintsfaq>  <https://uk.sagepub.com/en-gb/eur/press/sage-publishing-launches-advance-a-social-sciences-preprints-service-in-partnership-with> |
| Springer and Springer Nature | Posting of preprints is not considered prior publication and will not jeopardize consideration at Springer journals. Manuscripts posted on preprint servers will not be considered when determining the advance provided by a study under consideration at a Springer journal.  (Springer Nature) Preprints may be posted at any time during the peer review process. Posting of preprints will not jeopardize being considered for publication in Springer Nature journals | [Preprint sharing (springer.com)](https://www.springer.com/gp/open-access/preprint-sharing/16718886)  [Preprints : Springer Nature Support](https://support.springernature.com/en/support/solutions/articles/6000258807-preprints) |
| Taylor & Francis | Preprints accepted. Policy recognises preprint has become a routine activity for researchers in many disciplines | <https://editorresources.taylorandfrancis.com/welcome-to-tf/policies-guidelines/preprints/> |
| Wiley | Wiley will consider review articles previously available as preprints. | [Preprints Policy \| Wiley](https://authorservices.wiley.com/author-resources/Journal-Authors/open-access/preprints-policy.html) |
| Key paper on 14 publishers’ policies on Preprints | Key paper on 14 publishers found in the scoping review (snowballing sample) | [Preprint policies among 14 academic publishers \| Elsevier Enhanced Reader](https://reader.elsevier.com/reader/sd/pii/S0099133318304270?token=1DE45CD689F1F2A4C2CB79713634330CF43959D438B30F037D27BD553CC5D62A730E6BC4E4862DD419A8699B8DAA5778&originRegion=eu-west-1&originCreation=20220915132157) |
