# Supplementary material for: The use and acceptability of preprints in health and social care settings: A scoping review
Source: PLoS One. 2023 Sep 15;18(9):e0291627. doi: 10.1371/journal.pone.0291627 (PMC10503772; doi:10.1371/journal.pone.0291627)
Supplement: S4 Table — (DOCX) [file pone.0291627.s006.docx]

**Table S4: Funding organisations position on preprints for grant applications and publications (in alphabetical order)**

| **Funding organisation** | **Published/ Funding** | **Notes on preprints** | **Source (last accessed 10/07/23)** |
| --- | --- | --- | --- |
| Australian Research Council | Both | The Australian Research Council (ARC) will allow the referencing and inclusion of preprints in any part of a National Competitive Grant Program (NCGP) grant application, both within the Research Outputs list as well as the body of an application. *“Preprints and other comparable resources will form part of a holistic assessment of research outputs undertaken by peer reviewers to consider the quality and novelty of proposed research.”* | [Adjustments to the ARC’s position on preprints \| Australian Research Council](https://www.arc.gov.au/news-publications/media/communiques/adjustments-arcs-position-preprints) |
| British Heart Foundation | Funding | An appreciation of the value of all research outputs – where appropriate applicants will soon be asked to provide information regarding relevant research outputs; including but not limited to, datasets, inventions, preprints, protected IP, influence in policy and clinical guidelines, in addition to publications. Reviewers and committee members are required to consider the value of all research outputs in their assessment of research productivity. | <https://www.bhf.org.uk/for-professionals/information-for-researchers/how-we-award> |
| Canadian Institutes of Health Research | Both | CIHR recognizes the importance of considering the value and impact of all research outputs in addition to research publications. CIHR accepts the inclusion of preprints in grant applications as interim research outputs.  Complete the common CV: Only include publications that have been accepted or are in press. Do not include publications in preparation or submitted (unless available in a recognized scientific public archive (e.g., preprints)). | [Preprints at CIHR - CIHR (cihr-irsc.gc.ca)](https://cihr-irsc.gc.ca/e/50574.html)  [Doctoral Research Awards – Application instructions - CIHR (cihr-irsc.gc.ca)](https://cihr-irsc.gc.ca/e/38887.html) |
| Cancer Research UK  (CRUK) | Both | Strongly encourage posting preprints in public preprint servers as this practice speeds up dissemination of research findings, gives credit to researchers who can claim their work early, and accelerates and improves scientific progress. In addition, CRUK encourages publication of datasets.  In applications, peer review and non-peer-reviewed citations are listed separately, and the latter require a DOI.  *CRUK publication policy indicates:* all CRUK-funded researchers are strongly encouraged to post preprints of their work en route to publication in a peer-reviewed journal, and to publish them under a CC-BY licence on a platform that is indexed in Europe PMC.  Information on preprints and funding can be found in the CRUK website describing the organization’s research funding process and how funding decisions are made (signatories to the San Francisco Declaration on Research Assessment (link is external) (DORA). | <https://news.cancerresearchuk.org/2017/05/30/we-accept-preprints-in-grant-applications-new-guidance-for-researchers/>  <https://www.cancerresearchuk.org/funding-for-researchers/applying-for-funding/policies-that-affect-your-grant/policy-on-open-access>  <https://www.cancerresearchuk.org/funding-for-researchers/applying-for-funding/peer-review> |
| Chan Zuckerberg  Initiative | Both | Overall, CZI supports platforms where researchers can quickly disseminate methods, tools, preprints, and other research outputs. They have partnered with organisations contributing to the infrastructure underlying open science. [Partners with ASAPbio (scientist-driven non-profit promoting innovation and transparency in the life sciences) to “drive the global adoption of preprints among life scientists and to promote the value of preprints and open research outputs by institutions and funding agencies”.](https://asapbio.org/mag-2022) CZI also partners with Cold Spring Harbor Laboratory in their mission to support bioRxiv and medRxiv. The initiative provided $2 million in funding to support medRxiv.  The CZI has made posting a preprint (at the same time as the paper is submitted to a journal) a requirement for its grantees. | [ASAPbio: Building Support for Preprints - Chan Zuckerberg Initiative](https://chanzuckerberg.com/science/programs-resources/open-science/sharingplatforms/asapbio-building-support-for-preprints/)  [Chan Zuckerberg Initiative Awards $2 Million to medRxiv, a Health Sciences Preprint Server and Top Source of Breaking COVID-19 Research - Chan Zuckerberg Initiative](https://chanzuckerberg.com/newsroom/2-million-to-medrxiv-top-source-breaking-covid-19-research/) |
| European Research Council | Funding | *" ...preprints can be listed as part of an applicant's track record - if they are properly referenced and either a link to the preprint or a DOI is provided. With this clarification the Scientific Council intends to recognise the role that preprints play in frontier science, especially when time is of the essence."* | [ERC plans for 2019: Over €2 billion for Europe's most daring research ideas \| ERC: European Research Council (europa.eu)](https://erc.europa.eu/news/erc-plans-2019-over-%E2%82%AC2-billion-europes-most-daring-research-ideas) |
| Gates Foundation | Publication | The Foundation Open Access Policy has a brief section on preprints where grantees are encouraged to deposit Funded Research consisting of their submitted manuscript, and its subsequent versions, on a preprint server under a CC BY 4.0 license. | <https://openaccess.gatesfoundation.org/open-access-policy/> |
| Helmsley Charitable Trust |  | The organisation actively encourages and supports preprints: *“In February 2017, ASAPbio received a $1 million grant from the Leona M. and Harry B. Helmsley Charitable Trust to develop a new service to aggregate life sciences preprints and promote their visibility and innovative reuse.”* | <https://philanthropynewyork.org/news/helmsley-charitable-trust-supports-new-service-aggregate-life-sciences-preprints>  <https://scholarlykitchen.sspnet.org/2017/04/18/stars-aligning-preprints/> |
| Human Frontiers Science Program (HFSP) | Both | Starting in 2017 applicants have been able to list preprint articles in the publication section of HFSP proposals. Awardees were also permitted to cite publications deposited in freely available preprint repositories in interim and final reports to the organisation. HFSP Applications guidelines indicate that they are a signatory to the San Francisco Declaration of Research Assessment (DORA) and evaluate research proposals based on content (not solely by the criterion of Journal Impact Factors (JIF)).  It is important to note that the guidelines include a statement about preprints requiring a DOI and that the number of publications in proposals will consider *“peer-reviewed papers including preprints on public servers, accepted publications and publications in press, but not manuscripts in preparation or submitted.”* However, the note on considering peer-reviewed papers, including preprints, does not fully meet the definition of preprints published on their main website: *“a preprint is a complete scientific manuscript that is uploaded by the authors to a public server. The preprint contains complete data and methodologies; it is often the same manuscript being submitted to a journal. After a brief quality-control inspection to ensure that the work is scientific in nature, the author’s manuscript is posted within a day or so on the Web without peer review … ”* | <https://www.hfsp.org/Use-of-Preprint-Servers>  <https://www.hfsp.org/sites/default/files/Sciences/Grants/LI%20Guidelines.pdf> |
| Marie Curie | Publication | Reference to Europe PMC guidance on preprint seems to indicate acceptance of this type of publication by Marie Curie, however, it is not clear whether all preprints’ servers are acceptable to the organisation or whether applicants or grant holders are able to use preprints citations during funding application or within their research publications and reports. The reference is found in the guidelines to comply with Marie Curie Open Access policy. | <https://www.mariecurie.org.uk/globalassets/media/documents/research/grants/marie-curie-oa-policy-202021.pdf> |
| National Health and Medical Research Council's (NHMRC) | Publication | Brief mention of preprints in document: *Publication and dissemination of research: a guide supporting the Australian Code for the Responsible Conduct of Research.* A statement on preprints is found in the section about researchers’ responsibilities to disseminate research findings. The statement indicates that researchers can post a research manuscript on public servers as a preprint and remain aware of their obligations to ensure *“that research findings are disseminated responsibly and accurately, and action taken to correct the record in a timely manner if necessary.”* (p5). PloS and preprints.org published guidance on preprints is referenced in the Code document. | <https://www.nhmrc.gov.au/sites/default/files/documents/attachments/publications/publication_and_dissemination_of_research_guide.pdf>  <https://www.preprints.org/how_it_works>  <https://plos.org/open-science/preprints/> |
| National Institute for Health and Care Research  (NIHR) | Publication | The NIHR offers researchers the NIHR Open Research platform, in a similar model to the one described for the Wellcome Trust. It is offered to researchers supported by the NIHR for rapid sharing of research results and facilitating scientific discussion. Articles previously posted on a preprint server, such as ArXiv, bioRxiv or PeerJ Preprints can be submitted for publication in NIHR Open Research. NIHR reserves the right to ensure the use of preprints in the context of emergencies, however, preprints are considered out of scope of the NIHR Open Access policy. | <https://openresearch.nihr.ac.uk/?gclid=EAIaIQobChMIo6744bu3-gIVU_l3Ch1vEQLeEAAYASAAEgL7N_D_BwE>  <https://openresearch.nihr.ac.uk/about/policies> |
| National Institutes of Health (NIH) | Both | March 2017: As described in the document *Reporting Preprints and Other Interim Research Products,* the NIH encourages investigators to use interim research products (i.e., complete, public research products that are not final), such as preprints and preregistered protocols, to speed the dissemination and enhance the rigour of their work.  Research products can be cited in CVs (biosketches), research plans, other project information forms, career development awards and fellowships supplemental forms. Citation of products must include a Digital Object Identifier and the Object type (e.g., preprint, protocol). Requirements help reviewers in understanding that the product is public, interim, and in identifying the specific version cited. Researchers should also ensure that the characteristics of servers (or repositories and platforms) where interim products resulting from NIH funded awards are deposited meet a set of criteria. | <https://grants.nih.gov/grants/guide/notice-files/NOT-OD-17-050.html> |
| Simons Foundation | Publication | Announcement encouraging scientists to post preprints  *“The Simons Foundation encourages PIs to post preprints on recognized servers, such as arXiv https://arxiv.org/ or bioRxiv http://biorxiv.org/, in parallel with (or even before) submission to a peer-reviewed journal. The Simons Foundation also encourages PIs to publish under Open Access licenses, which are allowable budget costs. All preprints and Open Access publications resulting from projects supported by the Simons Foundation must be submitted through proposal CENTRAL with the Annual and Final Progress Report web forms. Please note that the PI’s Professional Profile populates the Publication section where preprints are added.”* | <https://www.simonsfoundation.org/2016/06/20/foundations-announce-support-for-scientist-driven-effort-to-promote-use-of-preprints-in-the-life-sciences/>  <https://www.simonsfoundation.org/2016/08/22/scgb-supports-preprints/>  <https://www.simonsfoundation.org/funding-opportunities/policies-and-procedures/> |
| UKRI | Both | MRC welcomes the inclusion of preprints in publication lists contained within grant and fellowship applications, applications for posts that require evidence of research productivity, MRC unit and institute quinquennial review progress reports, and future proposals. Regardless of the mode of publication (OA or subscription-based journal), the MRC requires that results of funded research be archived in Europe PubMed Central (Europe PMC) and made freely available within six months of the first online publication. Sharing pre-peer-reviewed manuscripts (effectively preprints) is encouraged on recognized preprints servers (e.g., bioRxiv, PeerJ Preprints, arXiv, SocArXiv or PsyArXiv). Preprints can be cited in grant applications only if the output has a persistent identifier (e.g., DOI). In addition to allowing applicants to cite preprints in applications and CVs, authors are encouraged to cite preprints in publications, where appropriate. MRC’s open access policy does not apply to preprints but applies to any peer-reviewed paper.  UKRI confirmed commitment to the 2016 Statement on data sharing in public health emergencies by becoming signatory to the joint: *“in the context of a public health emergency of international concern, research findings are made available via preprint servers before journal publication, or via platforms that make papers openly accessible before peer review.”* | <https://www.ukri.org/about-us/mrc/our-policies-and-standards/research/preprints/> |
| Wellcome Trust | Both | News article, January 2017: Researchers to cite preprints, or pre-peer reviewed manuscripts, in their grant applications and end-of-grant review reports.  The organisation supports the inclusion of preprints in grant applications and end-of-grant reports. Publishing preprints before peer review is encouraged under a CC BY licence on platforms indexed in Europe PMC that support immediate publication of complete manuscripts. Preprints publications are a requirement in events of disease outbreak where there is significant public health benefit. To comply with their OA policy, Version of Record or the Author Accepted Manuscript should be made freely available in PMC and Europe PMC. Wellcome offers researchers the platform *Wellcome Open Research* to allow researchers publication of their research in a rapid manner, supporting transparency, reproducibility, and impact. | <https://wellcome.org/news/we-now-accept-preprints-grant-applications>  [Open Access Policy - Grant Funding \| Wellcome](https://wellcome.org/grant-funding/guidance/open-access-guidance/open-access-policy)  <https://wellcomeopenresearch.org/> |
| Considerations in publishing research outputs in the international context | Publication | Publication in Open Access models requiring cover of APCs presents challenges to countries where investment in research is limited. Some initiatives such as the Public Knowledge Project (PKP) in Vancouver, have tried to address the issue by encouraging use of preprints servers and making research publicly available. Supported by this initiative is the Diamond OA model with more than 130 research-supporting institutions across the world becoming signatories.  Platforms supporting the model: Érudit (non-profit publishing platform publishing research in humanities and social sciences and select physical and natural science journals) and Redalyc, an OA initiative for non-commercial Open Access of Science as a public good (awarded a grant from Arcadia, a charitable fund in London) with strong presence in Latin American countries. | <https://www.nature.com/articles/d41586-022-03201-w>  <https://preprints.scielo.org/index.php/scielo/preprint/view/4729/version/5022>  <https://www.scienceeurope.org/our-resources/action-plan-for-diamond-open-access/>  <https://www.erudit.org/en/>  https://www.redalyc.org/ |
