# Supplementary material for: The use and acceptability of preprints in health and social care settings: A scoping review
Source: PLoS One. 2023 Sep 15;18(9):e0291627. doi: 10.1371/journal.pone.0291627 (PMC10503772; doi:10.1371/journal.pone.0291627)
Supplement: S5 Table — (DOCX) [file pone.0291627.s007.docx]

**Table S5. Future considerations and recommendations for publishers, funding organisations and the research community**

| **Focus** | **No.** | **Recommendation** |
| --- | --- | --- |
| Funding organisations | 1 | Consider the positioning of preprints as part of the peer review process and a preprint mandate for all research funding organisations (preprints becomes embedded in the process – mandated under plan U)^(1),^ including how to deal with preprint versions prior to version of record (allow researchers to make changes to manuscripts submitted to preprints or only the final version (based on community feedback), especially in terms of results, conflicts of interest, funding sources -and perhaps authorship?)^(87)^ |
| Funding organisations | 2 | Research institutions and funding organisations need to develop more explicit and objective guidance for the performance evaluation of preprints and require peer reviewers (for both funding applications and publications) to adhere to the guidance so that fairness and impartiality is conducted during the reviewing process^(25)^ |
| Funding organisations | 3 | Consider accepting submissions to preprint servers with enhanced editorial checks to minimise the risk of disseminating preprints that are not appropriate. Consider situational factors that may influence the impact of some preprint’s submissions (when is it not appropriate to publish research findings on a preprint server/platform)^(8)^ |
| Publishers | 4 | Policy standardisation should be carefully crafted and monitored to increase confidence in the use of preprints by academics in science^(39)^ |
| Publishers | 5 | Appropriate preprint server recommendations based on discipline / field of research due to inconsistent policies between different journals within the same publisher, or policies that show changes over time. Fluctuating preprint policies within the same publisher also send mixed signals to potential authors about the stability of preprints, and gives the perception that there is still uncertainty^(70, 109)^ |
| Publishers | 6 | Clear caveats on any preprint submission ensuring that there are appropriate links to any version revisions, through to the published article^(4, 64)^ |
| Publishers | 7 | Where preprints are cited in peer-reviewed journal publications, they must be clearly indicated as such and remain under press embargo until published in a journal^(3)^ |
| Publishers | 8 | Journals and publishers should have a publicly available preprint policy. There are two critical challenges before accepting preprint submissions; a double-blind review is impossible because reviewers can find the manuscript and authors on preprint servers; and authors should be allowed to cite preprints to maintain a more accurate citation of their sources^(6)^ |
| Publishers | 9 | Raise awareness of the limitation of preprints and where it is in the lifecycle of publication for non-scholarly readers such as the public and the media^(12, 86)^ |
| Publishers | 10 | Consider a two-step publishing model. Authors share ideas/findings on preprint channels and then publish in journals. A model where journal editors invite submissions from Authors whose preprint articles have gained attention could be a cost-effective model for Open Access Green 2019^(3, 4, 102)^ |
| Publishers | 11 | Strict editorial policies mandating data sharing (where appropriate) as a condition of the publication process. Encourage all publishers and journals to examine their data availability policies and ensure they are sufficiently stringent and consistently enforced. Clear policies mandating authors to provide data sharing statements (where applicable)^(105)^ |
| Publishers | 12 | Clear guidance regarding authorship in preprints manuscripts are required to ensure transparency and openness from the outset. Option is to use the same standards as peer reviewed publications (e.g., CRediT) which could strengthen editorial procedures and evaluation, as well as the development of research within the publication process^(26)^ |
| Publishers | 13 | Funding sources, author competing interests, and involvement of funders in creating or deciding to post the preprint should be declared. For clinical trials, preprints should include reference to the trial’s registration and follow appropriate reporting guidance (e.g., CONSORT for clinical trials)^(92)^ |
| Publishers and Funding organisations | 14 | Accessibility and feasibility to submit and upload to preprint servers on behalf of the researcher/s (therefore ensuring continuity)^(62)^ |
| Publishers and funding organisations | 15 | Preprint servers must have clear ethical and retraction policies in place, and these must be enforced and considered in publishers and funding organisations preprint policies^(12)^ |
| Publishers and funding organisations | 16 | Preprints should not be posted when the reported information could be misapplied or misused, causing significant consequences to health and safety^(92)^ |
| Publishers and funding organisations | 17 | Preprint policies should include ethical guidance in the event of misconduct and consider whether this should be the same process as peer-reviewed journal articles^(29)^ |
| Publishers and funding organisations | 18 | Adopt open access standards to meet publishing and funder requirements to prevent predatory journals^(48, 91)^ |
| Publishers, Researchers and Funding organisations | 19 | Center for Open Science (COS) has led to over 60 journals adopting badges on published articles to indicate open data, data sharing, materials, and/or pre-registrations, and such signals could also be adopted by preprint services (with clear guidance)^(9, 12, 28)^ |
| Publishers, Researchers and Funding organisations | 20 | Assess and validate tools such as ATOPP (a 21-item questionnaire called Attitudes Towards Open data sharing, Preprinting, and Peer-review (ATOPP), which measures attitudes towards open data, preprint servers, open peer-review, and open peer-review in scientific communities). With funding organisations and institutions encouraging or mandating open science practices, validated tools could aid in the assessment and monitoring of researchers’ attitudes and their associations with open science practices^(110)^ |
| Publishers, Researchers and Funding organisations | 21 | Consider the external factors and global challenges regarding access to research findings such as budgetary constraints affecting institutions, early career researchers, LMICs etc. What is the current position of funding organisations and is there consistency on the approach taken? |
| Researchers | 22 | Clear guidance about what is a preprint (with examples) and the stages of preprints as this may not be fully understood by the research community, the public and media coverage (providing caveats on preprints, what they are and what they are not); apply a watermark stating that these are preprints. Information sharing process should make the distinction between a preprint and a peer-reviewed article. Preprints should be accessible in all forms (e.g., online, pdf) and marked as not peer reviewed or accepted for journal publication^(21, 92)^ |
| Researchers | 23 | Ensure researchers notify and update their preprint records accordingly and within a specified timeframe (including personal and institutional records such as ORCID)^(6, 64)^ |
| Researchers | 24 | Use of preprints to disseminate research requires further sensitisation and training is required among researchers to demystify some of the perceived challenges and hesitancy with preprints^(93)^ |
