# Supplementary material for: The use and acceptability of preprints in health and social care settings: A scoping review
Source: PLoS One. 2023 Sep 15;18(9):e0291627. doi: 10.1371/journal.pone.0291627 (PMC10503772; doi:10.1371/journal.pone.0291627)
Supplement: Attachment — Submitted filename: Reviewer comments and responses v0.3.docx [file pone.0291627.s008.docx]

| **No** | **Reviewer comments** | **Response** |
| --- | --- | --- |
| **Reviewer 1 comments** | | |
| 1 | The biggest element that I feel is missing from this review is the evidence that exists comparing preprints to their peer reviewed counterparts. This addition would particularly strengthen the argument against the notion that “preprints are substandard work” (lines 342-346). This is also relevant to the point made in Table 2 relating to “quality assurances and trustworthiness”. | Comparing preprints to its counterpart is reported throughout the paper on the specific areas of focus related to the research question. However, we note the relevance and importance of your comment, and we have strengthened the case where applicable between preprints and peer reviewed articles. As the purpose of the review was to explore and map the use and acceptability of preprints, rather than how they compare to, we are not in a position to change the direction of the review. However, we have teased out the evidence to address your comment. |
| 2 | Further commenting on the various preprint peer-review platforms would be a nice addition to reflect where the field is moving – over 6000 preprints have so far been reviewed in this manner and this is something that funders are increasingly considering. For a starting list of papers I think are missing or would add to this discussion; • Bero et al • Carneiro et al • Fleerackers et al • Klein et al • Pagliaro et al • Sumner et al • Nicholson et al These papers may have been excluded by the authors during their selection criteria but I do feel they would add to the impact of this review. | Thank you for these references. We have added the references where applicable. We have highlighted some of these platforms related to how publishers use them and which ones, and where applicable how funding organisations use them or what ones are acceptable (providing links to the relevant webpages). We have updated the tables S3 and S4 including additional notes that was not present in the original submission, highlighting the preprint position of publishers and funding organisations. We hope this improvement is sufficient to meet your comment.   Links with COVID and preprints (including suggested references) are reported in lines 375 onwards including 498). Due to the scope of the review these were excluded but we have picked up on the relevance in the conclusion/ discussion, so thank you for suggesting these references. |
| 3 | Limitations – a discussion on the limits with regards to the highly biased “Western science” literature. I note that the authors only included a single study focussed on the African continent. This is not as a result of the authors missing a large amount of literature – the literature simply is lacking for this region – but it’s a very important limitation and point of discussion. | Thank you for your comment. We have included a statement to note the potential regional bias (lines 520-527) |
| 4 | One of the major benefits of preprints is that they enable researchers to share and read work without paying APCs or access charges that they aren’t able to afford, thereby increasing equity in the publishing process. | Thank you. We have added your comment to the manuscript under "What are preprints?" (line 66-67) |
| 5 | The authors make numerous mentions to the importance and role of preprints during the COVID19 pandemic, referring to the pandemic as initiating a "cultural shift" in the use of preprints, which is direct reference to our work which has not been cited (Fraser et al, PLOS Biology) whereas a nature news piece (which took heavily from out work) has been cited in other places to support this statement. I think these statements should be supported with references to the literature and I'm happy for the authors to cite work other than my own as there are other studies that support this should they so choose. | Thank you for the direct link, and the reference has been included in the overall reporting of the impact of COVID on the role of preprints. |
| **Reviewer 2 comments** | | |
| 1 | As discussed in the paragraph on inclusion criteria, the review is restricted to “research disciplines that focused on health and social care”. This restriction is not mentioned in the title and abstract of the paper. Neither is it mentioned in the introduction and discussion sections. My recommendation is to mention this restriction in a much more prominent way. The restriction to the health sciences is important because many of the concerns about preprinting reported in the review are probably less relevant in disciplines outside the health sciences. | We have added the focus in the title of the manuscript, in the abstract, introduction and as a limitation in the discussion. |
| 2 | “A preprint is a scientific manuscript or report that has not been submitted to a peer reviewed journal”: I don’t think this is an accurate way to define a preprint. Typically an article is first posted as a preprint on a preprint server and then submitted to a journal. According to the definition of a preprint given by the authors, an article posted on a preprint server can no longer be called a preprint when it is submitted to a journal. This is confusing. My suggestion is to define a preprint as a scientific manuscript or report that is shared publicly before it is submitted to a journal. | Thank you - we have revised the text to say: Preprints are open and accessible scientific manuscripts that is shared publicly, through a preprint server, before being submitted to a journal (or a requirement of the journal policy to submit the manuscript to a preprint server as part of that process). |
| 3 | “The search strategy aimed to locate both published and unpublished studies”: I find this confusing. An unpublished study is not public and therefore cannot be located using a search strategy that considers only publicly available information. I think the authors mean ‘peer-reviewed and non-peer-reviewed studies’ instead of ‘published and unpublished studies’. | We have changed this to reflect your comment - peer-reviewed and non-peer reviewed articles |
| 4 | “Two databases were searched: Scopus and Web of Science on 24 August 2022”: The use of Web of Science and Scopus means that preprints were excluded from the search strategy. Web of Science nowadays indexes preprints in the Preprint Citation Index, but this index was not yet available in August 2022. Scopus indexes preprints, but only in a highly restricted way. (Preprints are visible only in author profiles in Scopus, not in the general search feature.) I believe the exclusion of preprints needs to be mentioned and motivated. The authors could have used other databases, such as Dimensions, that are more comprehensive and that also index preprints. The exclusion of preprints might lead to a bias, since some proponents of preprinting may have published their work only as a preprint, not as an article in a peer-reviewed journal. | Thank you. We have added as a limitation and have stated a need for further research in the future recommendations section (including more disciplines). However, the grey literature searching was conducted to draw on these 'missing' areas, which was why we conducted a systematic scoping review to be inclusive of all evidence and not just studies. |
| 5 | “There are also several funding organisations that allow the inclusion of preprints as part of a grant application, which are starting to be disseminated in published literature. However, the evidence was sparse”: The authors claim that evidence on support for preprinting by research funders is ‘sparse’. However, in 2022, a substantial number of funders expressed support for reviewed preprints. See https://www.coalition-s.org/statement-on-peer-reviewed-publications/ (note that this statement uses the term ‘publication’ instead of ‘preprint’) and https://elifesciences.org/inside-elife/ebadb0f1/elife-s-new-model-funders-support-use-of-reviewed-preprints-in-research-assessment. Is there a specific reason why this evidence from the gray literature was not included in the review? | Thank you for providing additional links. The first link has been included to the manuscript but would not have been picked up in the grey literature as this was only directed at funding organisations or publishers. However, Plan U was mentioned in the literature, so we have included Plan S and cOAlition S as part of the wider landscape.   The second link would not have been picked up as it was December 2022. However, the article and funding organisations listed (Gates foundation and Wellcome) were included in the purposive sampling. |
| 6 | “the absence of peer review has led to the withdrawal of several preprints”: I don’t understand this. Many peer-reviewed articles are also withdrawn or retracted. I don’t think it is correct to say that the absence of peer review leads to the withdrawal of a preprint. If the same article were peer reviewed by a journal, its flaws might be overlooked in the peer review process and it might be accepted for publication in the journal. The article might then subsequently be withdrawn. It is therefore difficult to determine whether the absence of peer review is the cause of a withdrawal. | We have amended the paragraph to clearly explain what we mean here. |
| 7 | “preprints can become part of the publication process”: I don’t understand this. In my understanding, preprinting is by definition part of the publication process. When a researcher posts an article on a preprint server, the article is made public and is therefore published. | We have amended the sentence to make it clearer what we mean by publication process - to clarify we mean published in a journal article, not published on a server as some publishers do not mandate it being on a server. We are suggesting that preprint servers become the realisation and mandated as part of the process to publish your research openly and accessible (for anyone to review) whilst it is also being peer-reviewed by those chosen by the journal you have submitted to, to publish in.  I hope this clarifies the point you raised. |
| 8 | “with caveats and cautionary measures to maintain quality, credibility, transparency and openness”: It is hard to disagree with this, but doesn’t the same also apply to articles in peer-reviewed journals? As the large numbers of retractions of articles in peer-reviewed journals demonstrate, many low-quality articles are published in these journals. Caveats and cautionary measures to maintain quality and credibility are needed not only for preprints, but also (and perhaps even more) for peer-reviewed journals. | We have added some additional text around this sentence to be inclusive of all forms of publication |
| 9 | “there was a consensus of a lack of original research to clearly articulate how and in what circumstances preprints adds value and provides tangible benefits to researchers, publishers, and funding organisations”: I don’t understand this. It seems to me that a lot of work has already been done on this, as shown in Table 3. | A broader perspective has been taken to clearly articulate what we were trying to say. |
| 10 | “Adopting the use of preprints in grant applications requires clear and ethical guidance to prevent retraction”: I can see the benefits of clear and ethical guidance, but I don’t see how this relates to retraction. Please elaborate. | Agreed. We have taken "to prevent retraction" out |
| 11 | “Adopt open access standards to meet publishing and funding organisations requirements to prevent predatory journals, who often get researchers to sign over their copyright to the research, and do not disclose author fees”: This is about predatory journals. I don’t see how this relates to preprinting. | We have deleted this from the manuscript |
| 12 | “The published version (peer reviewed publications) are already seen as part of the formality, and preprints could be an important addition to that scholarly communication.”: In my understanding, a preprint is itself a published version. See my earlier comment on this terminological issue. | We have deleted this sentence and expanded the previous one. As we have explained this on a previous comment and made changes to the manuscript, we felt that this did not need to be repeated again. |
| 13 | Finally, while this paper correctly points out the need to be cautious with preprints, especially in the health sciences, I think the discussion is not sufficiently balanced, since the paper doesn’t mention that peer-reviewed journal articles are facing similar challenges. Articles in peer-reviewed journals also need to be handled cautiously, since we know that many of them are unreliable (as shown for instance by the retraction of high-profile COVID-19 articles and by the large numbers of retractions of fraudulent articles in peer-reviewed journals). To make the paper more balanced, my recommendation is to make readers more aware (in the introduction and the discussion section) of the challenges faced by peer-reviewed journal articles. | Thank you for your comment. We have now addressed this in the introduction and discussion, highlighting that some challenges are not unique to preprints. |
| **Journal and Editor requirements and comments:** | | |
| 1 | Please ensure that your manuscript meets PLOS ONE's style requirements, including those for file naming. The PLOS ONE style templates can be found at  https://journals.plos.org/plosone/s/file?id=wjVg/PLOSOne_formatting_sample_main_body.pdf and  https://journals.plos.org/plosone/s/file?id=ba62/PLOSOne_formatting_sample_title_authors_affiliations.pdf | The manuscript has been updated in line with PLOS ONE's style requirements |
| 2 | Thank you for stating the following financial disclosure:   "This research was funded by the National Institute for Health and Care Research Coordinating Centre (NIHRCC), based at the University of Southampton, through its Research-on-Research Programme. The views and opinions expressed in the discussion are those of the authors and do not necessarily reflect those of the Department of Health and Social Care."   Please state what role the funders took in the study. If the funders had no role, please state: "The funders had no role in study design, data collection and analysis, decision to publish, or preparation of the manuscript."  If this statement is not correct you must amend it as needed.  Please include this amended Role of Funder statement in your cover letter; we will change the online submission form on your behalf. | The financial statement has been amended, thank you  We have also added this in our covering letter |
| 3 | We note that you have stated that you will provide repository information for your data at acceptance. Should your manuscript be accepted for publication, we will hold it until you provide the relevant accession numbers or DOIs necessary to access your data. If you wish to make changes to your Data Availability statement, please describe these changes in your cover letter and we will update your Data Availability statement to reflect the information you provide. | All data is provided in the supplementary material files. There is no other data to share. Relevant links to OSF have been added to the data and material section of the paper and a revised statement in the covering letter. |
| 4 | As it is the PLOS policy of not accepting (subjective) reviews but systematic reviews, it might be better to resubmit this manuscript in a revised form as a systematic review. I do not see that the manuscript includes enough original research for a research article. However, you may feel free to provide your reasons for resubmitting as a research article in a cover letter if you prefer to do so. In any case, please carefully consider the comments of the reviewers and provide point-by-point responses in a rebuttal letter. | Thank you for your comment. We have responded to your comment in our covering letter and have revised the paper to submit as a systematic review. |
