# Supplementary material for: The use and acceptability of preprints in health and social care settings: A scoping review
Source: PLoS One. 2023 Sep 15;18(9):e0291627. doi: 10.1371/journal.pone.0291627 (PMC10503772; doi:10.1371/journal.pone.0291627)
Supplement: Attachment — Submitted filename: Reviewer comments and responses v0.4.docx [file pone.0291627.s009.docx]

| **No** | **Reviewer comments** | **Response** |
| --- | --- | --- |
| **Reviewer 2 comments** | | |
| 1 | “(or a requirement of the journal policy to submit the manuscript to a preprint server as part of that process)”: This is not entirely clear. Consider rephrasing this, for instance as follows: “(or at the time of submission to a journal, as part of the submission process of the journal)”. | Thank you for the suggestion, this has been changed in the revised version |
| 2 | “(such as a report)”: I am not sure what the authors mean by this. It might be better to delete this. | This has been deleted |
| 3 | “a challenge felt by those submitting a peer reviewed journal article to a publisher”: This is slightly confusing, since at the time of submission to a publisher peer review typically has not yet taken place. My suggestion is to rephrase this, for instance as follows: “a challenge for articles published in a peer reviewed journal”. | Thank you for the suggestion, this has been changed in the revised version |
| 4 | “since the COVID-19 pandemic, different approaches to peer reviewing, preprints are starting to be increasingly considered by publishers along with funding organisations”: This sentence is unclear and needs to be fixed. | Thank you, the sentence has been changed to the following:  Existing evidence from Brierley *et al.* (2021) and Watson (2022) for example have highlighted how preprints do not differ substantially from peer reviewed journal articles and since the COVID-19 pandemic, there has been a notable increase in the number of preprints. |
| 5 | “the absence of peer review of manuscripts or reports submitted to a preprint server has led to the withdrawal of several preprints”: Like in my earlier review, I object against this statement. There is no way to know whether peer review would have identified the problems in a manuscript and would have prevented the need for the manuscript to be withdrawn. Ref. 3 also doesn’t seem to provide support for the authors’ claim. | Thank you, the sentence has been revised to note your comment:  Although the COVID-19 pandemic has informed a greater appreciation for the scholarly landscape on the use and acceptability of preprints, there have been withdrawals of several preprints.^(3)^ However, there is no way of knowing whether peer review would have identified the problems resulting in the withdrawal of the manuscript at the point of publication. This is also true for peer reviewed journal articles, as articles are only withdrawn or retracted once published (issues raised by readers or the authors themselves).  The references have been amended to reflect the comment. |
| 6 | “However, it is important to note that peer reviewed journal articles can also be withdrawn or retracted due to oversight during the peer review process conducted by the journal itself (editors choosing the peer reviewers).”: This sentence is unclear. Retraction or withdrawal of an article typically is NOT “due to oversight during the peer review process conducted by the journal itself”. It happens after peer review has been completed and after an article has been published in a journal, and it typically happens as a result of concerns raised by readers or by the authors themselves. | See above comment that addresses this point. |
| 7 | “(not all publishers stipulate processing your manuscript on a preprint server prior to, or as part of the submission process to a journal)”: This is not clear. Please clarify. | Thank you, the sentence has been revised to note your comment:  (not all journals ask authors to submit their manuscript on a preprint server as part of the submission process to a peer reviewed journal). |
| 8 | “the types of discrepancies are common between both preprints and a peer reviewed journal article”: It is not clear what types of discrepancies this is about. | These have been added to the revised manuscript such as  (e.g., study characteristics, outcome reporting, numerical and statistical values) |
| 9 | “Due to the limited evidence found in the review on the acceptability and use of preprints in grant applications, research is needed to clearly understand what position funding organisations are taking and how it is perceived and accepted by the research community”: I am not sure whether it is correct to say the evidence is ‘limited’. In my earlier review, I made the suggestion to add a reference to the following statement by cOAlition S: <https://www.coalition-s.org/statementon-peer-reviewed-publications/>. The authors state that they have included this reference in their revised paper. However, I cannot find the reference. I would like to suggest again to add the reference, since it provides an important example of the increasing level of support from funders for preprinting. | The reference (111) has been included to the statement which is a different web link as the one provided is no longer valid or accessible. |
| 10 | “(using other platforms such as Dimensions)”: I don’t see the relevance of the reference to Dimensions in this sentence. My suggestion is to delete it. | Thank you, this has been deleted |
| 11 | “Preprints are therefore not a substitute for peer reviewed journal publications”: I agree that preprints are not a substitute for peer reviewed publications, but I disagree that a peer reviewed publication needs to be a publication in a journal. Increasingly peer review is also performed outside the context of journals (e.g., Peer Community In). | Thank you for your comment, the sentence has been revised to note your comment:  The role of preprints is merely part of the process to reaching a peer reviewed publication. Preprints are therefore not a substitute for peer reviewed journal publications (and not all peer reviewed publications are published in a journal, and peer review can happen outside the context of journals such as Peer Community in) but form part of the overall system and process of disseminating research rapidly using openly accessible routes to advance scientific discovery. |
